# Autophagy-related mechanisms for treatment of multiple myeloma

**DOI:** 10.20517/cdr.2023.108

**Published:** 2023-12-25

**Authors:** Gül Kozalak, Ali Koşar

**Affiliations:** ^1^Faculty of Engineering and Natural Science, Sabancı University, Istanbul 34956, Turkey.; ^2^Center of Excellence for Functional Surfaces and Interfaces for Nano Diagnostics (EFSUN), Sabancı University, Istanbul 34956, Turkey.; ^3^Turkish Academy of Sciences (TÜBA), Çankaya, Ankara 06700, Turkey.

**Keywords:** Autophagy, multiple myeloma, unfolded protein response, bone marrow microenvironment, drug resistance, hypoxia, DNA repair and transcriptional regulation, apoptosis

## Abstract

Multiple myeloma (MM) is a type of hematological cancer that occurs when B cells become malignant. Various drugs such as proteasome inhibitors, immunomodulators, and compounds that cause DNA damage can be used in the treatment of MM. Autophagy, a type 2 cell death mechanism, plays a crucial role in determining the fate of B cells, either promoting their survival or inducing cell death. Therefore, autophagy can either facilitate the progression or hinder the treatment of MM disease. In this review, autophagy mechanisms that may be effective in MM cells were covered and evaluated within the contexts of unfolded protein response (UPR), bone marrow microenvironment (BMME), drug resistance, hypoxia, DNA repair and transcriptional regulation, and apoptosis. The genes that are effective in each mechanism and research efforts on this subject were discussed in detail. Signaling pathways targeted by new drugs to benefit from autophagy in MM disease were covered. The efficacy of drugs that regulate autophagy in MM was examined, and clinical trials on this subject were included. Consequently, among the autophagy mechanisms that are effective in MM, the most suitable ones to be used in the treatment were expressed. The importance of 3D models and microfluidic systems for the discovery of new drugs for autophagy and personalized treatment was emphasized. Ultimately, this review aims to provide a comprehensive overview of MM disease, encompassing autophagy mechanisms, drugs, clinical studies, and further studies.

## INTRODUCTION

Multiple myeloma (MM) is associated with the proliferation of a single plasma B cell variant in the bone marrow and the secretion of monoclonal immunoglobulins^[1]^. MM is the second most common type of hematological malignancy worldwide, which accounts for approximately 10% of cases^[2]^. The mean age of diagnosis is 69 years, and the disease diagnosis is based on the monoclonal M protein produced by malignant B cells^[3]^. Myeloma cells are plasma cells that secrete immunoglobulins and usually synthesize IgG or IgA^[4]^. As plasmacytomas increase, monoclonal gammopathy of undetermined significance (MGUS), a premalignant and painless disease, emerges. Subsequently, asymptomatic smoldering myeloma (SMM) and eventually symptomatic MM appear. 1% of cases with MGUS and 10% of cases with SMM might convert to MM^[5]^. Rapidly accumulating malignant B cells cause bone destruction, anemia, hypercalcemia, and renal failure^[6]^. In the clinical treatment of the disease, proteasome inhibitors, steroids, monoclonal antibodies, DNA damage, and immunomodulatory compounds are used in combination^[7]^. Most of these therapies focus on killing MM cells. Commonly, there are three different death mechanisms in cells: apoptosis (type 1), autophagy (type 2), and necrosis (type 3). Apoptosis eliminates irreparable cells and prevents damage from spreading. Nuclear fragmentation, chromatin condensation, and apoptotic bodies commonly crop up in cells during apoptosis^[8]^. Autophagy is a process of cellular self-degradation that helps the body repair itself by breaking down damaged materials under conditions of starvation or stress. In the autophagic mechanism, gathered proteins in the cell, wrecked organelles or microorganisms are digested in large vesicles with the help of the lysosome^[9]^. Necrosis is a critical condition that occurs when living tissue is damaged beyond repair, leading to cell death. An exogenous factor such as injury provokes necrosis formation in cells, which causes disruption of cell morphology and damage to organelles^[10]^. Currently, it was reported that cell death types can be converted into each other through different gene expressions^[11]^. For example, caspase-8 has a critical function as a bridge between the apoptosis and necrosis pathways^[12]^. Additionally, inhibition of autophagy was associated with necrosis, inflammation, and accelerated tumor growth^[13]^. This review extensively delves into the mechanisms of autophagy related to MM disease progression or regression. We clarified how signaling pathways and genes in autophagy mechanisms may affect the disease. Additionally, we elucidate how the drugs used to regulate autophagy affect the regulation of these genes. Finally, we offer valuable insights into specific mechanisms that should be targeted with appropriate drugs to leverage autophagy for the treatment of MM disease.

### The mechanisms of autophagy

**Figure 1:**
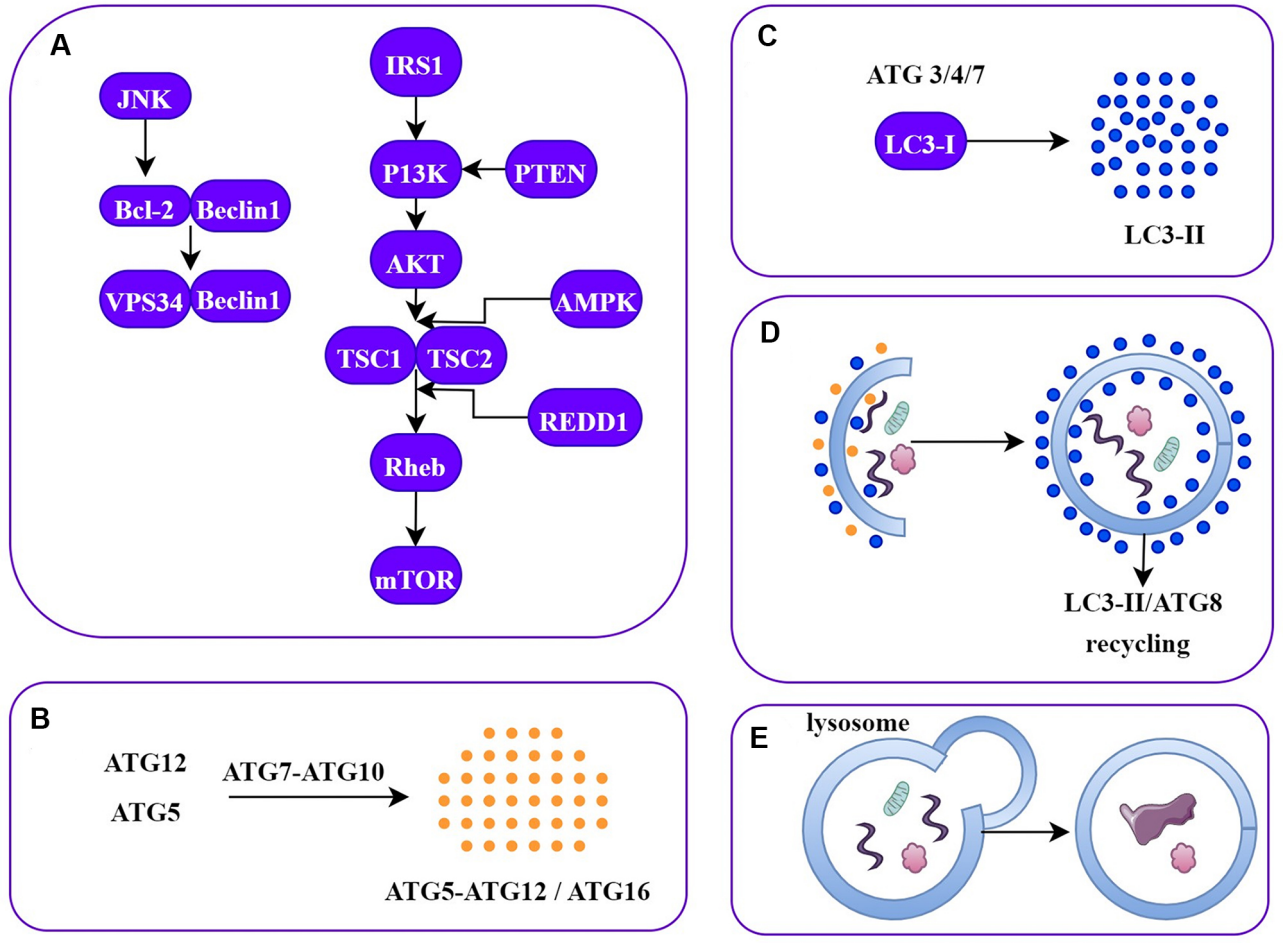
Schematic of the stages of autophagy. (A) Phagophore formation triggered by Beclin-1/VPS34 in response to a stress signal; (B) Multimerization resulting from ATG5/ATG12 conjugation and interaction with ATG16; (C) Embedding in the phagophore membrane by LC3 engraving; (D) Autophagosome formation by ATG4-directed LC3-II/ATG8 recycling; (E) Fragmentation of molecules following association with lysosome. ATG: Autophagy-related proteins.

Autophagy is a recycling activity that is employed to maintain the survival of other cells and to maintain body homeostasis in case of stress such as nutrient deficiency and reactive oxygen compounds (ROS)^[14]^. In tumorigenesis, autophagy can work in a bidirectional manner by increasing disease progression or causing cancer cell death^[15]^. In addition, differentiations in autophagic balance might lead to drug resistance in cancer cells. Commonly autophagy-related genes are under-expressed or contain deletions in cancer cells^[16]^. In recent years, it has been declared that the loss of PTEN, a tumor suppressor, leads to a decrease in autophagy and thus plays a key role in shaping the fate of tumor cells^[17]^. The formation of autophagosomes primarily serves as a recycling process, not exclusively aimed at cancer cells. However, it works in tandem with the immune system in its specific function related to cancer^[18]^. Many autophagy-related proteins (ATG) are involved in the regulation of autophagy. The mechanisms of autophagy can be summarized into five items^[19]^, as shown in Figure 1.

The initiation of phagophore formation is determined by the interaction between Beclin1 and Bcl-2, which is triggered by the phosphorylation of Bcl-2 by JNK-1. This results in the dissociation of Bcl-2 from Beclin1, which allows for the initiation of autophagy by the Beclin1/Vps34 complex. Recently, the role of Beclin1 and UV radiation resistance-associated gene (UVRAG) as a tumor suppressor and autophagy trigger in cancer was emphasized^[20]^. mTOR plays a pivotal role in regulating autophagy by inhibiting ATG1/ULK1-2 from the early stages. mTOR interacts with various proteins to form mTORC1/2 complexes, which activate different transduction pathways. Essentially, mTOR serves as the linchpin governing the balance between cell growth and the induction of autophagy. mTOR is inhibited by REDD1 and AMPK through Rheb under conditions of hypoxia or low ATP [Figure 1]. In addition, mTOR is activated via the IRS1, PI3K/AKT, TSC1/TSC2, and Rheb signaling pathways. Although three variants of autophagy have been proposed so far [microautophagy, chaperone-mediated autophagy (CMA), and macroautophagy], degradation by lysosomal fusion is inevitable. Macroautophagy is characterized by the formation of an autophagosome, which consists of lipid bilayers so that large proteins and organelles can be broken down and recycled by the lysosome^[19]^. In microautophagy, the cell membrane spontaneously folds inwards to form a tube. The end of the tube buds and connects with the lysosome through vesicles^[16]^. Unlike others, CMA is the selective degradation of misfolded proteins by lysosomes via chaperones. The KFERQ motif in misfolded proteins is recognized by Hsc70 and forms a complex^[21]^. This complex is pulled in and destroyed by LAMP-2A in the lysosome membrane without the need for vesicles. Finally, they are degraded into the fundamental units by lytic enzymes (protease, lipase, and hydrolase) in the lysosome and are released to the cytoplasm for synthesizing new complex molecules.

Autophagy is a crucial factor in the circumstance of the disease, as MM cells are plasma cells that synthesize a large amount of immunoglobulin. Therefore, it is important to assess how autophagy functions, considering its dual impact as both a suppressor and supporter of the tumor^[15]^. ATG5, an important autophagy-related indicator, is cleaved upon exposure to Bortezomib and initiates autophagy in MM cells^[22]^. It is known that inhibiting autophagy in conjunction with proteasome inhibitors, which are effective in treating MM, presents a promising therapeutic avenue. Therefore, it is necessary to elucidate the fundamental mechanisms of autophagy at the forefront of MM research. In this review, autophagy mechanisms and autophagy modulators in MM disease were comparatively reviewed. This review emphasizes the significance of incorporating autophagy alongside appropriate medications and timing as a strategy to significantly extend the lifespan of MM patients.

## AUTOPHAGY-RELATED MECHANISMS IN MM

MM cells use multiple mechanisms to evade cell death, including proteasome, unfolded protein response (UPR), and autophagy. MM cells that can escape from death develop resistance to drugs, which makes available therapies useless. The viability of MM cells is based on the proteasome-mediated degradation of misfolded proteins and non-functional newly synthesized proteins^[23]^. Therefore, autophagy can maintain the continuity of MM cells by averting the toxic stack of misfolded proteins. A basal level of autophagy is essential for the life cycle of MM cells. When these mechanisms collapse, apoptosis is finally induced by immune cells for the integrity and health of the organism^[18]^. Autophagy may be involved in the progression of MM from malignant plasma cells. Exemplarily, the enhancement of autophagy in plasma cells of MGUS and MM patients was only in the direction of MM^[24]^. A study conducted with biopsies of 89 patients with MM reported that patients with Beclin1 and LC3 biomarkers had a longer life expectancy^[25]^. It was revealed that elevated IFNγ in neutrophils isolated from MGUS and MM patients triggers JAK2/STAT3 signaling to form an autophagic survival system^[26]^. Recently, CD46, IKBKE, PARK2, ULK4, ATG5, and CDKN2A were considered in a three-cohort meta-analysis study that investigated the contribution of polymorphisms in autophagy-related genes to MM disease risk^[27]^. Considering all these, autophagy is related not only to MM progression or regression but also to MM development at the beginning. We can examine the mechanisms thanks to the fact that autophagy is effective in MM under the headings of UPR, bone marrow microenvironment (BMME), drug resistance, hypoxia, DNA repair and transcriptional regulation, and apoptosis.

### UPR

The folding of proteins takes place in the endoplasmic reticulum (ER). Misfolded proteins are ubiquitinated by a system including ER chaperones and are degraded by the proteasome^[28]^. Besides contributing to the degradation of p62/SQSTM1 aggregated proteins via proteasomes, it is known that p62 can sustain cell survival by inducing autophagy^[29]^. Additionally, recent studies have shown that SQSTM1/p62 plays a crucial role in supporting proteostasis and is not just involved in working with the proteasome to degrade Ub-protein aggregates^[30,31]^. SQSTM1/p62 is a vital component of the autophagic reserve that helps the cell adapt to proteasome stress induced by proteasome inhibitors with the modification of the interactome^[30]^. In this sense, it can be interpreted that p62 functions in the regulation of autophagy. Autophagy plays a significant part in the transformation of B cells into plasma cells, and ATG5 mediates these tasks^[32]^. Mice with Atg5^f/f^ were observed to have a higher ER stress with increased immunoglobulin synthesis compared to wild type^[33]^. Therefore, autophagy plays a role in guiding plasma cells to the bone marrow for adaptive immune responses. Myeloma cells are under constant ER stress due to the high content of immunoglobulins they synthesize and can easily induce the UPR^[34]^. Inhibition of the proteasome with chemotherapeutics makes the damaged proteins roll up, turn toxic, and trigger UPR^[35]^. UPR restores homeostasis and performs in conjunction with autophagy. Thus, autophagy is upregulated and initiates a survival mechanism to eliminate UPR.

The UPR proteolytic system is controlled by the signaling mechanism of IRE1/XBP-1, PERK/ATF4, and ATF6/cleaved ATF6. IRE1, PERK, and ATF6 are separated from GRP78, to which they are attached when UPR is triggered, which accounts for a signal transduction cascade^[36]^. The PERK signaling pathway conduces to the UPR system by suppressing protein synthesis. IRE1 participates both by augmentation of misfolded protein impairment and by enhancement of protein folding together with ATF6. The function of XBP-1 is of great importance in the autophagy mechanism in UPR and MM cells. IRE1 fulfills the cell viability role of autophagy by regulating itself and XBP-1 at the mRNA level^[37]^. Directing the maturation of plasma cells, XBP-1 is involved in autophagy through ATG5^[38]^. However, XBP-1 facilitates IRE1 binding to the BiP chaperone to downregulate the UPR function in B cells^[39]^. IRE1a/XBP-1 balance is critical in MM cells as it reduces ER stress^[40]^. Furthermore, it was reported that inhibition of XBP-1 splicing by IRE1 may be a treatment modality for MM cells^[41]^. Additionally, it was demonstrated that there is a correlation between elevated spliced XBP-1 levels and Bortezomib resistance^[35]^. XBP-1 is also involved in the regulation of ATG5 and Beclin1 via eIF2AK3 for the activation of autophagy^[42]^. Moreover, XBP-1 functions as a transcription factor in the signaling cascade between IRE1 and ATF6 cleavage in UPR^[43]^. XBP-1 influences the expression of genes grave in B cell differentiation, such as BAFF, APRIL, BCMA, and TACI^[44]^. In addition, these genes also affect NF-κB expression. In MM cells, the NF-κB pathway leads to the expression of cytokines, cell adhesion molecules, and cell growth and survival factors. NF-κB, which also mediates XBP-1 expression, contributes to the induction of UPR and autophagy in cancer cells^[45]^. It was previously mentioned that Beclin1 and LC3 work together in autophagosome formation. NF-κB raises the possibility of autophagy by augmenting Beclin1 expression^[46]^. PERK obstructs the phosphorylation of eIF2B, which leads to a significant reduction in the production of ATF4 and protein synthesis^[47]^. This mechanism serves as a crucial intervention point for regulating protein synthesis in cells. PERK can operate to diminish the oxidative stress response that may be motivated by unfolded proteins by activating NRF2^[48]^. In this sense, it plays a binary part in up or down-regulating UPR in autophagy. PERK labors with ATF4/6 to activate the transcription factor CHOP and to reduce ER stress^[49]^. From this point of view, PERK primarily functions to sustain cell life, but it can also operate cell death mechanisms in response to the strong signals it receives. In an experiment study where IRE1, ATF6, and PERK were individually knocked out using RNAi, it was indicated that PERK functions as a sensor of autophagy in MM cells^[50]^. An inhibitor that targets the PERK pathway via eIF2AK3 in MM cells holds great potential in inducing cell death and providing effective therapy^[51]^. This approach must be further explored to unlock its full potential in treating MM.

### BMME

**Figure 2:**
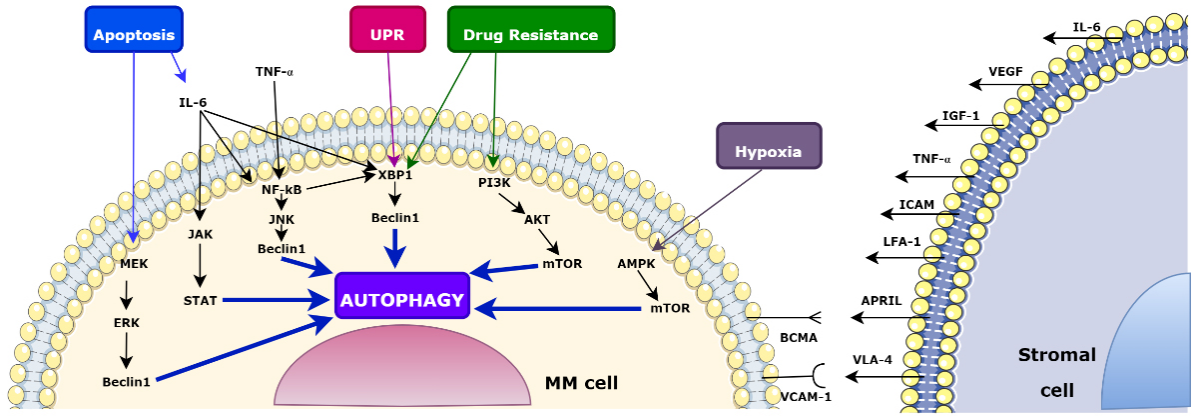
Causes and consequences of autophagy in MM cells within BMME. In the bone marrow, stromal cells secrete IL-6, VEGF, and IGF-1 due to intercellular interactions with MM cells. IL-6 stimulates UPR and autophagy through XBP-1. Apoptosis triggers autophagy via the JAK/STAT3 and MAPK/ERK pathways or IL-6. Autophagy and survival are promoted by the interaction of BCMA and APRIL through NF-κB and XBP-1. Adhesion molecules such as TNF-α, ICAM-1, LFA-1, and VLA-4 develop drug resistance by inducing autophagy via NF-κB. PI3K/AKT/mTOR inhibition promotes autophagy in drug-resistant cells. UPR upregulates HIF1-α through XBP-1 processing, which triggers autophagy through AMPK and mTOR. BMME: Bone marrow microenvironment; MM: multiple myeloma; UPR: unfolded protein response.

The BMME consists of cells and supporting elements. BMME stimulates a reputable part in the differentiation of B cells and their transformation into cancer^[52]^. As a result of the interaction of MM cells with stromal cells, IL-6, VEGF, and IGF-1 are secreted, and signaling pathways that cause tumor aggression, such as PI3K/AKT/mTOR, MEK/ERK, JAK/STAT3, and NF-κB, are activated [Figure 2]. Intercellular interactions between stromal cells and MM cells in the bone marrow and relationships with extracellular matrix (ECM) elements determine the fate of MM cells. Furthermore, adhesion molecules such as TNF-α, ICAM-1, LFA-1, and VLA-4 generated by stromal cells are responsible for the interaction of MM cells and bone marrow stromal cells (BMSCs). Stimulation of adhesion molecules for expression is mediated by NF-κB^[53]^ and ultimately contributes to the development of drug resistance in MM cells. As another factor, IL-6 is an interleukin released from stromal cells and contributes to the clinical prognosis, apoptosis, drug resistance, and metastasis processes of MM^[54]^. Elevated IL-6 levels induce XBP-1 generation, which results in the enhancement of UPR and autophagy^[24]^. APRIL and BAFF ligands, which bind to BCMA secreted from B cells, promote XBP-1-mediated autophagy via NF-κB, assisting in the survival of MM cells^[55]^. It is argued that there may be crucial mechanisms for cancer-associated fibroblasts (CAFs) in autophagy in cancer cells^[56]^. CAFs from Bortezomib-resistant patients were observed to protect MM cells from apoptosis in the co-culture^[56]^. Because autophagy is induced by the inhibition of TGF-β in Bortezomib-resistant CAFs, apoptosis is prevented by reducing ER stress^[57]^. Moreover, it was stated that mature adipocytes in BMME contribute to the induction of autophagy by MM cells to avoid apoptosis and to the development of drug-resistant phenotypes^[58]^. Autophagy also supports the immune system by taking part in the phagocytosis of microbes. LC3, IFN1, MHC class 2, and IL-6 aid in this process^[5]^. Apart from these, cancerous cells necessitate a higher nicotinamide adenine dinucleotide (NAD+) cycle and rely on the Nampt enzyme for regulation. The Nampt inhibitor FK866 suppresses the protective effects of BMME and demonstrates its effect in both resistant MM cell lines and MM patients^[59]^. Moreover, FK866 induces autophagy in MM through inhibition of mTORC1 and ERK1/2^[59]^. The involvement of the WNT signaling pathway in the self-renewal abilities and metastasis processes of MM cells has also been proven in another study^[60]^. Furthermore, it was found that exposure of MM cells to UV triggers the WNT pathway and induced autophagy^[61]^.

Angiogenesis is an important element in the transition of the disease from MGUS to MM^[62]^. Although it is a distinguishing fact that angiogenesis occurs only in MM, various cytokines play a significant role in the process^[63]^. Pro-angiogenic cytokines are released by interactions among MM cells, stromal cells, and endothelial cells. Studies reported that MM cells secrete higher levels of bFGF, HGF, and VEGF than plasma cells, which promotes angiogenesis^[64]^. In a recent study, it was concluded that endothelial cells from patients with MM expressed higher levels of EGFR and its ligand HB-EGF than those from MGUS patients^[65]^. When EGFR is activated, it promotes blood vessel growth and assists endothelial cells in surviving. Blockade of HB-EGF-EGFR signaling limited the angiogenic potential of bone marrow endothelial cells and hampered tumor growth in an MM mouse model^[65]^. These findings suggest that HB-EGF-EGFR signaling could be a potential target in anti-angiogenic therapy for MM. Another study aimed to investigate the effect of combining Bortezomib and Hydroxychloroquine on plasma and endothelial cells isolated from patients with MM and MGUS^[66]^. The results showed that the combined treatment had different effects on MM cells and endothelial cells^[66]^. LC3B and p62 expressions induced autophagy in MM cells^[66]^, and it was suggested that Bortezomib should be used in combination with an anti-angiogenic drug to enhance its effectiveness. Considering the dual role of autophagy in MM, it initially acts as a tumor suppressor, but may also promote tumor growth, survival, and therapy resistance. The relationship between EGFR and autophagy is intricate. In fact, inhibiting EGFR could trigger autophagy as a survival mechanism. Thus, by gaining a deeper understanding of the connection among EGFR, autophagy, and bone marrow angiogenesis, we can potentially develop novel treatments for MM.

### Drug resistance

Drug resistance is one of the major issues in MM and causes relapse and refractory of the disease. MM cells can avoid the toxic effects of chemotherapeutics via autophagy and thus develop drug resistance^[14]^. Bortezomib, which is frequently used in the treatment of MM and ratified by the FDA, triggers autophagy via UPR by inhibiting proteasomes^[67]^. Bortezomib resistance occurs frequently in MM, and different approaches are sought for treatment. Caspase-10 endorses the survival and resistance of MM cells by regulating autophagy^[15]^. Through induction of IRF4 in MM, caspase-10 cleaves BCLAF1, which competes with Beclin-1 for binding to Bcl-2 and inhibits autophagy^[68]^. Therefore, the sensitivity of MM cells to Bortezomib can be restored by developing caspase-10 inhibitors. The association of Bortezomib resistance with P-gp and drug resistance-related proteins in MM cells was demonstrated^[69]^. The increase in ATF4 expression after Bortezomib treatment in breast cancer cells^[70]^ suggests that Bortezomib resistance can be overcome by combined suppression of autophagy and proteasomes. In a recent study on Bortezomib-resistant MM patients and cell lines, it was mentioned that upregulation of CMA may be a mechanism leading to resistance formation and targeting this mechanism could be a therapeutic target^[71]^. It was revealed that the CMA pathway was induced due to the ER stress caused by Bortezomib, and the expression of LAMP2A, which is an indispensable part of this pathway, was boosted^[71]^. However, it was stated that inhibition of the CMA pathway restored Bortezomib sensitivity, and the dual combination was more cytotoxic^[71]^. It was revealed that the Profilin-1 gene contributes to Bortezomib resistance in MM by boosting autophagy through Beclin1^[72]^. The combination of anti-β2M monoclonal antibodies with Bortezomib in MM cells promoted cellular death even in resistant cells by inhibition of autophagy^[73]^. Bortezomib response decreased in MM cells as a result of suppression of NEDD4L, which induced autophagy by binding the 19S proteasome^[74]^. A mechanism for the NEK2/USP7/Beclin-1 complex was identified in autophagy-induced Bortezomib resistance in MM patients^[75]^. As a result of Bortezomib treatment applied to MM cells, autophagy was incited by GRP78 in cells^[76]^. Furthermore, it was displayed that HSPA5 expression was upregulated in Bortezomib-resistant cells^[76]^. It was declared that transfection of mir145-3p into MM cells suppresses HDAC4 and mTORC1 and causes sensitivity to Bortezomib due to autophagy^[77]^.

Autophagy functions in the development of resistance to many chemotherapeutics used in the treatment of MM. In a related study, by using primary patient samples and Dexamethasone-resistant MM cell lines, Beclin1 and LC3/ATG8 expressions were found to be inversely proportional to the p62 level^[78]^. In this case, the authors claimed that autophagy leads to drug resistance in MM, and Beclin1/p62 is a biomarker. Additionally, antiestrogen binding site ligands regulated the cholesterol mechanism via the PI3K/AKT/mTOR pathway, inducing autophagy and overcoming Dexamethasone resistance^[79]^. The relationship of Carfilzomib resistance with autophagy in MM cells and the contribution of the KLF4 transcription factor were obvious^[80]^. KLF4 attaches to the p62 gene promoter and upregulates its expression. It was emphasized that Doxorubicin-induced resistance in MM cells is mediated by DEPTOR, and autophagy plays a main role in this resistance^[81]^.

### Hypoxia

Tumor cells have a high metabolic rate and require large amounts of nutrients and oxygen. The needs of the rapidly growing tumor mass cannot be met at the same rate through the vessels, and a hypoxic environment develops. Cancer cells can continue to proliferate by inducing HIF1-α and adapting to these unfavorable conditions. HIF1-α triggers autophagy by inhibiting Bcl-2/Beclin1 and upregulating ATGs through BNIP3 and its ligand^[82]^. However, tumor cells can initiate autophagy independently of HIF1-α through the activation of mTOR or AMPK^[83]^. Since BMME has a low pO_2_ level compared to blood, MM cells can easily adapt to the hypoxia environment^[84]^. The hypoxia state around the MM cell provokes the induction of UPR and autophagy due to the aggregation of the unfolded protein^[85]^ because many proteins need O_2_ in order to be folded properly. Due to hypoxia-induced O_2_ deficiency, sufficient ATP cannot be produced from glucose. In this case, MM cells can also induce starvation-induced autophagy^[86]^. It was reported that upregulation of Hexokinase-2 in hypoxia conditions intensifies autophagy in MM cells and introduces it as an antiapoptotic feature^[87]^. UPR initiates hypoxia by upregulating the expression of HIF1-α and VEGF via XBP-1^[88]^. In addition, activation of mTORC1, an autophagy-related factor, was found to increase susceptibility to hypoxia in MM cells^[89]^.

### DNA repair and transcriptional regulation

Dysfunctional DNA repair pathways facilitate the progression and metastasis of MM disease from its initial stages to the development of drug-resistant phenotypes, because autophagy can be induced through DNA repair mechanisms^[90]^. The activation of autophagy via the mTORC1/TSC2/ULK1 pathway is triggered by the ATM-mediated phosphorylation of AMPK^[91,92]^. Additionally, ATM enables the inhibition of mTOR through the activation of Che-1 via REDD1 and DEPTOR^[93]^. Furthermore, stress-induced Sestrin2 binds to GATOR2, thereby suppressing mTORC1^[94]^. NF-κB, also activated by ATM, leads to the activation of Beclin1 and induction of autophagy^[95]^. ROS-induced DNA damage assists in the suppression of AMPK and triggering of autophagy by activation of PARP1^[96]^. ATM can also induce autophagy through the regulation of p53. p53 induces the initiation of autophagy through the upregulation of PTEN and AMPK^[97]^. Moreover, DAPK regulated by p53 facilitates autophagy through its connection with Beclin1 phosphorylation and VPS34 activation^[98]^.

Histone deacetylases (HDACs) are crucial transcription regulators and can control functional proteins in signaling pathways. HDAC6 is critical in MM due to its connection with UPR. Because of its deficiency, autophagy can be induced by forming aggresomes. Therefore, it is a promising target for patient treatment. HMGB1 is a DNA-binding protein that contributes to the functionality of nucleosomes and causes tumor progression. HMGB1 expression is increased during the treatment of various cancers with different chemotherapeutics^[99]^. HMGB1 is involved in autophagy and DNA damage repair, and the knockdown of HMGB1 in MM cells restores mTOR-mediated Dexamethasone sensitivity^[100]^. Additionally, it was reported that HMGB1 expression increased in Bortezomib-resistant MM cells, and the combination of Bortezomib and Lycorine reversed the resistance^[101]^. Furthermore, it was emphasized that MALAT1, a lncRNA, caused the upregulation of HMGB1 in MM cells, and the knockdown of MALAT1 caused a decrease in the expressions of Beclin1 and LC3B together with HMGB1 in MM^[102]^. In light of these findings, it is clear that targeting HMGB1 in drug-resistant MM patients is an essential and promising therapeutic approach.

### Apoptosis

Apoptosis against cellular stress is the first type of cell death pathway and is used to eliminate situations where autophagy cannot be overcome. Depending on the stress state of the cell, these death mechanisms can block or trigger each other. Apoptosis in MM cells can be induced by IL-6, VEGF, IGF1, SDF1, and FGF factors via the JAK/STAT3 and MAPK/ERK pathways^[16]^. It was reported that drug-resistant MM cells induce apoptosis via the Apo2L/TRAIL pathway^[103]^. The expression of Mcl-1, a proliferation marker of MM cells, increased by JAK/STAT3 and VEGF, resulting in resistance to apoptosis^[104]^. The importance of the Bcl-2/Bax ratio in MM apoptosis is indisputable^[105]^, and the increase in Bcl-XL protein leads to the inhibition of apoptosis via IL-6. NF-κb’s involvement in MM cell apoptosis, metastasis, and drug resistance was investigated^[106]^. Autophagy helps to alleviate metabolic stress, but when apoptosis and autophagy are both impaired, it can lead to necrotic cell death^[13]^. Therefore, autophagy plays a role in suppressing tumors by reducing metabolic stress and preventing necrotic cell death, along with apoptosis^[13]^.

The similarity of proteins in the apoptosis and autophagy pathways is a well-known phenomenon in MM, which highlights the complex interplay between these cellular processes^[18]^. As an example, Beclin1 executes its function as a tumor suppressor through regulation by AMBRA1, Bif1, and UVRAG. Both siRNA-mediated inhibition of Beclin-1 and autophagy inhibition by 3-methyladenine and Chloroquine resulted in apoptosis of MM cells^[107]^. Furthermore, inhibition of autophagy with 3-methyladenine augmented Oridonin-dependent apoptosis by regulating ROS and SIRT1 in MM cells^[108]^. Beclin1 inhibition extinguishes both autophagy and apoptosis, because Bcl-2 inhibits apoptosis by MOMP inhibition and stops autophagy by tethering Beclin1 or AMBRA1^[109]^. It was claimed that HMGB1 disturbs the equilibration between Beclin1 and Bcl-2 in the resistance to Bortezomib in MM^[101]^. AMBRA1 induces autophagy and suppresses apoptosis^[110]^. UVRAG induces autophagy by activating Beclin1, while Bif1 induces tumor suppression via autophagy^[111]^. In addition, a high expression of p38/MAPK engenders autophagy, while a low expression causes apoptosis^[112]^. STAT3 regulates many autophagy-related genes, such as FOXO1/3, by sequestering eIF2AK2 to fulfill this function^[113]^. It was suggested that the nutritional status marker mTORC1 could be responsible for drug resistance in MM via the AKT/ERK signaling pathway^[114]^. In a study with MM patients and cell lines, it appeared that gene expressions related to the PI3K/AKT/mTOR pathway and autophagy were higher in resistant groups^[115]^. Additionally, it was also unveiled that inhibition of PI3K/AKT/mTOR signaling promotes autophagy and apoptosis^[115]^. Autophagy can also induce apoptosis by aggregation of caspase 8^[116]^. Although p53 is an important biomarker in cancer, its role in the control of cell cycle and apoptosis in MM was clarified^[105]^.

Recent studies reveal that MM cells can reshape cellular traffic mechanisms to combat ER stress. FAM46C, which is mutated in more than 10% of MM patients, is frequently emphasized. In a study on MM cells, it was reported that reactivation of FAM46C triggered apoptosis and ER stress by forming a complex with FNDC3A, an ER-associated protein^[117]^. The FAM46C and FNDC3A complex increases the exocytosis of lysosomes, which causes changes in cellular traffic and secretion^[117]^. This leads to aggregate accumulation and negative regulation of autophagy in MM cells^[117]^. Eventually, MM cell-specific FAM46C/FNDC3A-mediated tumor suppression occurs by induction of apoptosis^[117]^. Another study highlights the tumor suppressor and therapeutic role of FAM46C^[118]^. FAM46 interaction with FNDC3 proteins stabilizes ER-targeted protein mRNAs and increases ER growth and Ig secretion^[118]^. However, when the proteasomal balance is disrupted, p62 associates with FAM46C and prevents its interaction with FNDC3 proteins^[118]^. The p62/FAM46C/FNDC3 ensures the survival of MM cells, and targeting this pathway with proteasome inhibitors appears as a mechanism that enables selective cell death.

## THERAPEUTICS FOR AUTOPHAGY IN MM

Autophagy plays a crucial role in the development of MM, a disease where plasma cells produce large quantities of immunoglobulin. Combining autophagy inhibition with proteasome inhibitors is an effective MM treatment. Since autophagy can work in both directions, strategies to induce or inhibit autophagy could be helpful in the treatment of MM. Benefits can be obtained by inhibiting the tumor mass-preserving effect of autophagy or by directly triggering autophagy in MM cells. Therefore, suppressing the PI3K/AKT/mTOR signaling pathway to induce autophagy in MM cells may be a therapeutic option. The PI3K/AKT/mTOR signaling pathway is associated with p38/MAPK, PTEN, p53, SIRT1, IGF-1 and ROS. Since the MAPK/ERK signaling pathway is in connection with mTOR, its suppression in MM may inhibit autophagy. JNK may have a tumor suppressor task due to its capacity to regulate the function of Beclin1. Ca^2^+ itself, as a signaling molecule, can trigger autophagy through its ability to activate Beclin1 and AMPK. The above-mentioned signaling pathways can be regulated with drugs to achieve the desired autophagy benefit in MM. For this reason, Table 1 lists drugs that can inhibit or induce autophagy by various mechanisms, especially in MM. Table 2 provides an overview of the most promising autophagy modulators clinically tested in MM patients.

**Table 1:** Autophagy modulators and functioning mechanisms in MM

**Mechanism**	**Drug**	**Effect**	**Ref.**
AMPK activators	Metformin	Activation	[119-121]
	Spermidine		[122]
AMPK and mTOR inhibitors	Resveratrol	Activation	[123,124]
Autophagy flux inhibitors	Elaiophylin	Inhibition	[125]
	4-Acetylantroquinonol B		[126]
Autophagy inducer	Tat-Beclin1 peptide	Activation	[127]
BTK inhibitors	Ibrutinib	Activation	[128]
Ca^2^-ATPase inhibitors	Thapsigargin	Inhibition	[129,130]
Class I PI3K inhibitors	CH5132799	Activation	[131]
	GDC-0941		[132]
Class III PI3K inhibitors	3-Methyladenine	Inhibition	[107,108,133,134]
	Wortmannin		[135]
	LY294002		[136]
HDAC inhibitors	Vorinostat	Activation	[137,138]
Lysosomal alkalizers	Chloroquine	Inhibition	[107,139,140]
	Hydroxychloroquine		[66,134]
	Lys05		[141]
Lysosome membrane permeabilization	Thymoquinone	Inhibition	[142]
mTORC1/2 inhibitors	Rapamycin	Inhibition	[124,134]
	Everolimus		[143,144]
	Deforolimus		[145]
	Temsirolimus		[146]
	AZD8055	Activation	[147]
	Torin1		[148]
	PP242		[149]
PI3K/AKT inhibitors	Perifosine	Activation	[150]
PI3K/mTOR inhibitors	Apitolisib	Activation	[151]
Protease inhibitor	Pepstatin A	Inhibition	[152]
	E64d		[68,153]
Vacuolar H-ATPase inhibitors	Bafilomycin A1	Inhibition	[140,154]
	Concanamycin A		[155]
VPS34 kinase inhibitors	Paclitaxel	Inhibition	[156,157]

MM: Multiple myeloma.

**Table 2:** Summary of autophagy therapeutics in MM clinical trial

**Title**	**Therapeutics**	**Phase**	**Publication**	**Identifier**
A pilot study of infusional Cyclophosphamide and pulse Dexamethasone with Rapamycin or Hydroxychloroquine in patients with relapsed or refractory multiple myeloma	Hydroxychloroquine Rapamycin	Early phase 1	-	NCT01396200
A Phase I/II trial of Hydroxychloroquine added to Bortezomib for relapsed/refractory myeloma	Bortezomib Hydroxychloroquine	Phase 1	[158]	NCT00568880
A Phase I dose escalation study of Hydroxychloroquine with infusional Cyclophosphamide, pulse Dexamethasone, and Rapamycin in patients with relapsed or refractory multiple myeloma	Cyclophosphamide Dexamethasone Hydroxychloroquine Sirolimus	Phase 1	-	NCT01689987
A Phase II, trial of Chloroquine in combination with VELCADE and Cyclophosphamide in patients with relapsed and refractory myeloma	Velcade Cyclophosphamide Chloroquine	Phase 2	[139]	NCT01438177
A Phase 1B/2 multicenter, open label, dose-escalation study to determine the maximum tolerated dose, safety, and efficacy of ACY-1215 (RICOLINOSTAT) in combination with Pomalidomide and low-dose Dexamethasone in patients with relapsed and refractory multiple myeloma	Ricolinostat Pomalidomide Dexamethasone	Phase 1 Phase 2	-	NCT01997840
A Phase 1/2, open-label, multicenter study of ACY-1215 administered orally as monotherapy and in combination with Bortezomib and Dexamethasone for the treatment of relapsed or relapsed/refractory multiple myeloma	Ricolinostat	Phase 1 Phase 2	[159]	NCT01323751
A Phase 1/2, open-label, multicenter study of ACY-1215 (Ricolinostat) in combination with Lenalidomide and Dexamethasone for the treatment of relapsed or relapsed/refractory multiple myeloma	Ricolinostat Lenalidomide Dexamethasone	Phase 1	[160]	NCT01583283
An open-label phase I study of the safety and efficacy of RAD001 in combination with Lenalidomide in the treatment of subjects with relapsed and relapsed/refractory multiple myeloma	Everolimus Lenalidomide	Phase 1	[161]	NCT00729638
Phase II trial of RAD001 in relapsed/refractory multiple myeloma	Everolimus	Phase 2	-	NCT00618345
An open-label phase 1 study of Metformin and Nelfinavir in combination with Bortezomib in patients with relapsed and/or refractory multiple myeloma	Bortezomib Metformin Nelfinavir	Phase 1	-	NCT03829020
Phase II trial, open label, clinical activity of Metformin in combination with high-dose of Dexamethasone (HDdexa) in patients with relapsed/refractory multiple myeloma	Metformin Dexamethasone	Phase 2	[120]	NCT02967276

MM: Multiple myeloma.

### Lysosomal alkalizers

Lysosomes are important organelles of autophagy, and changes in pH levels prevent the activity of lysosomal enzymes and the fusion of autophagosomes with lysosomes. As mentioned before, the life of MM cells is dependent on the protein synthesis cycle. Therefore, the combination of lysosomal alkalizers and proteasome inhibitors or alkylating agents is a powerful therapeutic option for the selective killing of MM cells. It is known that Hydroxychloroquine, which is widely used in the treatment of malaria, can inhibit autophagy in cancer cells^[162]^. The combination of Bortezomib and Hydroxychloroquine increased cellular cytotoxicity via autophagy in MM patient samples^[66]^. In the Phase 1 study of MM patients, the same treatment combination not only improved disease symptoms but also reduced side effects^[158]^. In Phase II trials, whose Chloroquine was administered in combination with Bortezomib and Cyclophosphamide, a curative effect was shown for relapsed and refractory MM patients^[139]^.

### HDAC inhibitors

The performance of the HDAC6 inhibitor Panobinostat added to the MM treatment protocol in Phase 3 trials is clear^[163]^ because HDAC6 inhibitors work together with UPR in toxic protein aggregation to promote autophagy. Additionally, HATs and HDACs can control the acetylation status of autophagy-related genes. An example of HDAC inhibitors inducing autophagy is the inactivation of mTOR by Vorinostat. Autophagy is suppressed by phosphorylating ULK1 through mTOR. The HDAC inhibitor Vorinostat triggers autophagy in tumor cells through the production of ROS^[164]^. The combination of Suberoylanilide hydroxamic acid (Vorinostat), 17-allylamino-17-demethoxy-geldanamycin (Tanespimycin), and Clonazepam overcame the complication of peripheral neuropathy by inducing autophagy via HSP70 or LAMP-2A^[165]^. Moreover, the efficacy of the combination of Clarithromycin, a high-spectrum antibiotic, in MM and various cancers has been previously reported^[166]^. Clarithromycin’s suppression of autophagy by reducing cytokines such as IL-6, which is an important factor in MM development and drug resistance, makes it a possible candidate. It was shown that the combination of Bortezomib, Lenalidomide, and Clarithromycin would also be suitable for therapy without Dexamethasone in MM patients with diabetes^[167]^. The combination of Vorinostat, Bortezomib, and Clarithromycin in MM cells causes cell death and upregulation of CHOP genes that stimulate ER stress^[137]^. In this sense, combinations of proteasome inhibitors and next-generation HDAC6 inhibitors may serve as therapeutic options to increase autophagy in MM cells.

### Vacuolar H-ATPase inhibitors

Vacuolar type H-ATPases are pumps responsible for regulating intracellular organelle pH. Inhibiting these pumps with specific inhibitors blocks autophagy and prevents cell metastasis. The combined effect of Bortezomib and Bafilomycin A1 in MM cells is more cytotoxic than their use alone, which reveals the link between UPR, autophagy, and ER stress^[154]^. Furthermore, a combination of Ixazomib and autophagy inhibitors Bafilomycin A and Chloroquine increases cytotoxicity and cell death via JNK^[140]^. Therefore, the combination of vacuolar-type H-ATPase inhibitors with proteasome inhibitors stands as a potential therapeutic option. Additionally, inhibition of autophagic flux is also a treatment option. In a recent study, the treatment of p53 mutant MM cells with Elaiophylin suppressed autophagy and resulted in cell death by apoptosis^[125]^*.*

### AMPK activators and mTOR inhibitors

AMPK plays a critical role in responding to stress caused by metabolic disorders. It helps in maintaining the balance between anabolic and catabolic reactions, which is crucial for the stability of the cell and energy preservation. In cancer treatment, AMPK activators are used to trigger autophagy and cell death. In peripheral neuropathy, one of the best-known side effects of Bortezomib treatment in patients, pathological changes occur in Schwann cells. Metformin, which is used as an antidiabetic drug, in combination with Bortezomib, suppressed GRP78 and promoted autophagy^[120]^. Furthermore, Metformin was recently shown to induce apoptosis and necrosis in MM cells and mouse models^[119]^. Additionally, Metformin applied to MM cell lines and mouse models promoted cell proliferation arrest and autophagy by AMPK and mTORC1/2 regulation^[121]^. Thus, AMPK activators are a promising option for inducing autophagy by selectively targeting MM cells.

mTOR plays a cornerstone role in autophagy by regulating proliferation and metabolism. Resveratrol induces cell cycle arrest, apoptosis, and autophagy. In a study conducted to prove this, it was emphasized that Resveratrol induces autophagy and apoptosis in MM cells by inhibiting AMPK and mTOR^[123]^.

### PI3K/AKT/mTOR inhibitors

The PI3K/AKT/mTOR signaling pathway promotes the survival and proliferation of tumor cells. Therefore, inhibition of one or more proteins on the signaling pathway causes tumor regression. A report on testing autophagy-regulating drugs in MM cells stated that Rapamycin can induce apoptosis and autophagy, Hydroxychloroquine can inhibit autophagy and induce apoptosis, and 3-methyladenine can only inhibit autophagy^[134]^. Furthermore, the combination of 3-methyladenine and VEGF inhibitor Bevacizumab in MM cells promoted apoptosis^[133]^. In a recent study, NVP-BEZ235 was discovered to induce autophagy through the mTOR2-Akt-FOXO3a-BNIP3 signaling pathway in MM cell lines, mouse models, and primary samples^[168]^. Targeting the PI3K/AKT/mTOR signaling pathway, which is central to cell life and death choices, is a good strategy and a powerful therapeutic option for clinicians.

### Other drugs or compounds

Glycosphingolipids located outside the cell membrane are important in cell adhesion, cell-cell interactions, and oncogenesis. In MM and MGUS preclinical models, it was shown that Eliglustat can inhibit autophagy through the glycosphingolipid mechanism, thereby maintaining osteoclastogenesis and preventing bone loss^[169]^. The tumor burden-reducing effect of the combination of hexokinase-2 inhibitor 3-bromopyruvate and Bortezomib was published^[87]^. It was reported that Solamargine alone induces autophagy, and its combination with Bortezomib may be a good treatment strategy^[170]^. The induction of autophagy in MM cells by inhibitors of the isoprenoid biosynthesis pathway is a potential target for developing new therapeutic drugs^[171]^.

Dihydroartemisinin induced autophagy and apoptosis via WNT/β-catenin and P38/MAPK in MM cell lines and mouse models^[172]^. Hsp90 inhibitor 17-dimethylaminoethylamino-17-demethoxygeldanamycin triggered autophagy over mTOR inhibition in MM cells^[173]^. Clioquinol engendered autophagy by causing mTOR downregulation in MM and leukemia cells^[174]^.

Trifluoperazine, an antipsychotic drug, caused the inhibition of autophagy by NUPR1 overexpression in MM cells and led to the induction of apoptosis^[175]^. Betulinic acid performs its cytotoxicity in MM cells by inducing autophagy or apoptosis via the PP2A switch^[176]^. It was reported that Fingolimod regulates apoptosis by inducing autophagy in MM cells^[177]^.

### Drug discovery

Currently, there is no known treatment option to be considered definitive for targeting autophagy in cancer. This is because traditional cell cultures do not fully describe the complex interaction between cancer cells, other cells, and the microenvironment. However, 3D cell cultures are more effective in depicting the tumor microenvironment than traditional cultures, and they better reflect the differentiation and proliferation of tumor cells^[178]^. Microfluidic systems are biomedical platforms that enable the controlled distribution of small amounts of liquids, which facilitates the simultaneous execution of multiple tests^[179]^. These devices allow for more accurate and quicker studies of the tumor microenvironment, metastasis, and drug resistance^[1]^. They also enable drug screening to be carried out rapidly and precisely, which assists in identifying new drug candidates with more efficiency^[180]^.

## CONCLUSION

MM is a disease of plasma cells and is very sensitive to protein turnover processes. Autophagy, a mechanism linked to cell death, contributes to the progression of the disease by activating the UPR system in MM cells. Proteasome inhibitors, commonly employed in MM, induce UPR and ER stress. However, inhibiting proteasomes not only triggers autophagy but also leads to drug resistance. Therefore, there is a close interplay among UPR, autophagy, apoptosis, and drug resistance. The combination of proteasome inhibitors such as Bortezomib and Carfilzomib, which are frequently used in the treatment of MM, and autophagy inhibitors can effectively induce cell death. Additionally, agents that inhibit heat shock proteins also show therapeutic potential in MM by initiating autophagy. Furthermore, as in all cancer processes, autophagy in MM is affected by BMME, DNA repair, and transcriptional regulation, crucial aspects in the development of MM disease. The addition of autophagy inhibitors to agents that cause DNA damage will prevent MM cells from evading death. The widespread use of HDAC6 inhibitors in treating MM cells deactivates the UPR protection and induces autophagy. Clinically, PI3K/AKT/mTOR inhibitors prove effective by restricting MM cell growth, making cell death inevitable. Furthermore, even in normal cells, stress-causing starvation and hypoxia conditions trigger autophagy. Consequently, VEGF and EGFR inhibitors, which will prevent MM cells from feeding, and HIF1-α inhibitors, which will cause oxygen deprivation, become beneficial. Moreover, small molecules that target genes involved in the autophagy mechanism also have a therapeutic potential. There is a considerable volume of both completed and ongoing clinical trials exploring this avenue. However, a clinically promising drug has not yet been released, and there is no standardized autophagy treatment yet. In this regard, leveraging 3D and microfluidic systems holds the potential to develop disease models. These platforms have the capability to generate results more rapidly than traditional clinical trials. Additionally, reconstructing disease models with 3D models and microfluidic systems can lead to the identification of new autophagy modulators^[1,178]^. Moreover, it is possible to conduct tests on new agents before administering them to the patient and to develop appropriate strategies that can be tailored according to the patient's needs. This enables the utilization of autophagy, similar to apoptosis, in halting or even eradicating the progression of MM.

## DECLARATIONS

### Copyright

© The Author(s) 2023.
